# Risk and Protective Factors Associated With Support of Violent Radicalization: Variations by Geographic Location

**DOI:** 10.3389/ijph.2021.617053

**Published:** 2021-03-29

**Authors:** B. Heidi Ellis, Alisa B. Miller, Georgios Sideridis, Rochelle Frounfelker, Diana Miconi, Saida Abdi, Farah Aw-Osman, Cecile Rousseau

**Affiliations:** ^1^ Department of Psychiatry and Behavioral Sciences, Boston Children’s Hospital and Harvard Medical School, Boston, MA, United States; ^2^ Department of Psychiatry, McGill University, Montréal, QC, Canada; ^3^ School of Social Work, University of Minnesota, St. Paul, MN, United States; ^4^ Canadian Friends of Somalia, Ottawa, ON, Canada

**Keywords:** violent radicalization, discrimination, mental health, North America, Somali

## Abstract

**Objectives:** We examine the association between perceived discrimination, mental health, social support, and support for violent radicalization (VR) in young adults from three locations across two countries: Montréal and Toronto, Canada, and Boston, United States. A secondary goal is to test the moderating role of location.

**Methods:** A total of 791 young adults between the ages of 18 and 30, drawn from the Somali Youth longitudinal study and a Canada-based study of college students, participated in the study. We used multivariate linear regression to assess the association between scores on the Radical Intentions Scale (RIS) with demographic characteristics, anxiety, depression, social support, and discrimination.

**Results:** In the full sample, discrimination, age, and gender were associated with RIS scores. When we examined moderation effects by location, RIS scores were associated with depression only in Montréal, and with social support (negatively) and discrimination in Toronto. None of the variables were significant in Boston.

**Conclusion:** These findings suggest that an understanding of risk and protective factors for support of VR may be context-dependent. Further research should take into consideration local/regional differences.

## Introduction

Violent radicalization (VR) is a significant and growing threat worldwide [1, 2]. VR can be understood as “an individual or collective process whereby normal practices of dialogue, compromise, and tolerance between groups/individuals with diverging interests are abandoned and one or more groups/individuals engage in violent actions to reach a specific (political, social, religious) goal” [3]. In a context of increased social polarization, VR is affecting both majorities and minorities, targeting different forms of otherness: racial, ethnic, religious, political and gender-related (e.g., [4]). There is interest in understanding and addressing both proximal and distal risk factors that contribute to the process of VR [5]. This shift calls for a public health approach that entails distinguishing between primary, secondary, and tertiary prevention efforts [6]. Primary prevention constitutes a focus on determinants of VR opinions and attitudes in the general population, intending to prevent VR through community-based programming that emphasizes positive civic engagement, global citizenship, and constructive dialogue on polarizing issues [7].

Recent years have seen an increase in empirical work seeking to identify individual-level risk and protective factors related to VR. This research has produced inconclusive results. In a recent systematic scoping review of factors related to VR, relative deprivation of a social group—including grievances, injustices, victimization, and stigmatization—was the most common risk factor identified for radicalization to international extremism [8]. Experiences of discrimination have been linked to VR, particularly when those experiences are thought to be on the basis of language or political views [9, 10]. However, no relation between perceived discrimination and sympathy for VR was found in a sample of Muslims in England [11]. In addition, Somali youth and young adults who endorsed support for VR reported moderate, but not high, levels of exposure to discrimination in the North American context [12].

A range of psychological factors are associated with VR, some of them potentially operating as mediators between grievances and VR. Rousseau and colleagues (2019) report that depression accounts for 25% of the relationship between grievances (i.e., experiences of discrimination) and sympathy for VR [10]. Importantly, the exact association between mental health and VR is unclear; mental health symptoms—depression, in particular—are generally predictive of extremist attitudes only in conjunction with other factors [13]. These mixed findings may be partially explained by differences in study populations and local influences specific to each country.

A socio-ecological framework posits that micro, meso and macro-level risk and protective factors uniquely contribute to, and interact with one another, to produce vulnerability and resilience to radicalization [6]. As such, there is a need to move beyond a focus on individual-level risk factors and examine the relationship between larger sociopolitical contexts and support of VR. VR is a complex phenomenon that varies in expression based on the unique social, cultural, and historical contexts of diverse societies [14]. Such research entails identifying the contribution of area-level effects (such as cities, regions, and countries) on support and endorsement of radical ideology [15]. To date, empirical evidence exploring the role played by the larger social context is extremely limited, with some preliminary work indicating country and regional variations in risk factors for support of VR [16, 17].

### Context of Violent Radicalization in Toronto, Montréal and Boston

Discourse and events related to VR vary widely across different locales. In Canada, VR became a heated political issue after the aborted Toronto attacks associated with religious radicalization in 2006. In this incident, a group of 18 youth (known as the “Toronto 18”) was arrested on terrorism charges. These arrests led to negative portrayal of Muslims in the media and growing suspicion and mistrust of Muslims in public opinion. Miller and Sack [18] examined over 200 newspaper columns, opinions and letters to the editor in the aftermath of this event. They conclude that a “significant portion of the published commentary raised an unreasonable public alarm, cast suspicion on the followers of a major religion and impugned Islam itself” (p. 279). As one of the biggest and most visible Muslim groups in Toronto, Somalis were impacted by this event and its aftermath, especially as some of those arrested were Somali. In the province of Quebec (Canada), an attack by a lone actor in St-Jean and the departure of Montréal youth to join DAESH in Syria in 2015, brought VR under the spotlight in 2014. Subsequently, a deadly attack against a mosque in the city of Québec in 2017 highlighted the increasing attraction exerted by different extremist discourses (extreme right and religious) in youth. These events occurred as heated public debates about immigration and cultural and religious diversity highlighted the sharp divide between the very cosmopolitan city of Montréal and the relatively more homogenous rest of Québec. Although social polarization and the associated upsurge in extremist movements has been notable both in Toronto and in Montréal, the specific local forms of intercommunity tensions are shaped by different historical and social factors in both cities: the multiculturalism ideology in Toronto and the tensions around the French language and identity in Montréal.

Similarly, the United States has experienced extremist attacks from diverse ideologies including far right, far left, and religious radicalization [19]. Within Boston, the Boston Marathon bombings of 2013, perpetrated by two brothers who espoused religious extremist ideology, drew national and international attention. In the wake of these bombings, public discourse with in Boston united around the slogan of “Boston Strong,” a call for perseverance in the face of hurt, unity across religious and ethnic lines, and rejection of hate [20, 21], although some segments of society responded to the events with bias and discrimination such as believing Islam is more likely than other religions to encourage violence [22, 23] and recommending the profiling of young Muslim men [24].

Understanding common vs. unique risk and protective factors for VR between populations and geographical settings has critical implications for cross-cultural applicability of research findings and, ultimately, how local governments or communities seek to shape programs and policies to reduce VR in their regions. In this study, we seek to further this nascent body of research by examining the association between psychosocial risk factors and support for VR across different populations (Somali and general population youth) in three different urban cosmopolitan settings spanning two countries: Montréal and Toronto, Canada, and Boston, United States. Specifically, we examine the association of discrimination, depression, anxiety (risk factors), and social support (protective factor) with support for VR. We hypothesized that after controlling for age and gender, each of the above risk and protective factors would be associated with support for VR. We further hypothesized that patterns of associations would differ by city.

## Methods

### Population Studied

The following analyses utilize pooled data from the multi-site Somali Youth Longitudinal Study (SYLS) and a multi-site college study in Canada.

#### Boston and Toronto

Current analyses draw on data from 198 Somali young adults located either in Boston or Toronto who participated in Wave 2 (data collected between 2014 and 2015) of SYLS. SYLS eligibility included having lived in the United States or Canada for at least 1 year, either born in Somalia or being of Somali descent, and being between the ages of 18 and 30 at the time of initial enrollment. Multiple strategies including snowball sampling and spreading information about SYLS through community meetings were used to recruit participants.

#### Montréal

Students from fourteen colleges in Québec, Canada participated in a study on sympathy for VR from 2016 to 2017. Participants were eligible to participate if they were registered as full-time students in one of the participating colleges. The response rate varied greatly between the colleges, ranging from 2 to 19%. Only respondents in the greater Montréal area were included in this analysis (*n* = 593 from six colleges).

### Measures

#### Demographics Variables

Participants were asked to self-report demographic variables. Gender was self-reported as a dichotomous variable (male/female) in SYLS. Participants self-reported their gender as male, female or other in the college survey. Age was reported as a continuous variable in SYLS (how old are you?) and a categorical variable in the college survey (18, 19–21, 22–24, 25–27, 28–30, and 31+). For the purposes of these analyses, SYLS age data were coded into six categories to match the college study. The location of interview was recorded by SYLS staff and in the college study, participants identified the college they attended. All SYLS participants were of Somali ethnicity. In the college study, participants self-reported whether or not they (and their mother/father) were born in Canada and if not, asked to specify what region of the world they had been born.

#### Radicalism Intention Scale

The RIS is a four-item subscale of the Activism and Radicalism Intention Scales (ARIS; [25]; that measures an individual’s readiness to participate in illegal and violent behavior for one’s group or organization (support of VR). Respondents rate their agreement to statements on a seven-point Likert scale, ranging from 1 (disagree completely) to 7 (agree completely). A sample item is, “I would participate in a public protest against oppression of my group even if I thought the protest might turn violent.” Of note, an adapted version of the RIS was used for SYLS participants in order to increase acceptability within the Somali community. More specifically, items were rephrased to assess *attitudes towards someone who commits legal or illegal actions*, vs. *personal intentions* to commit these actions. A mean score was calculated with higher scores indicating more support for violent radicalization. Both the original ARIS and the adapted ARIS have demonstrated good psychometric properties [9, 25, 26]. Cronbach’s alpha for support of VR in this study were acceptable (alpha = 0.858; alpha = 0.861; alpha = 0.878) for Boston, Toronto, and Montréal respectively.

#### Discrimination

The Everyday Discrimination Scale (EDD [27]; is a nine-item measure of perceived discrimination, assessing day-to-day experiences of discrimination. Sample items include “being treated with less courtesy than other people” and “people act as if they think you are dishonest.” Seven options for frequency of occurrence provided ranged from “never” to “almost every day.” Responses were dichotomized into “never” and “occurred.” A mean score was calculated with higher scores indicating higher levels of discrimination. The EDD has demonstrated validity and reliability [28, 29]. Cronbach’s alpha for discrimination were good (alpha = 0.813; alpha = 0.866; alpha = 0.874) for Boston, Toronto, and Montréal respectively.

#### Mental Health

The depression and anxiety subscales of the Hopkins Symptoms Checklist (HSCL [30]; were used to measure mental health. The depression subscale is a 15-item subscale that measures symptoms and problems related to depression. For the current study, a 14-item depression subscale was used as one item (sexual interest) was removed in SYLS to increase acceptability; this item was dropped from the Québec data in the current analyses. The anxiety subscale consists of ten items measuring symptoms and problems related to anxiety. Respondents are asked to reflect on the past 4 weeks only and indicate how much they have had the identified symptoms or problems on a 4-point Likert scale, ranging from “not at all” to “extremely.” A mean score for each subscale was calculated with higher scores indicating more symptomology. The HSCL has demonstrated good psychometrics in immigrant groups [31]. Cronbach’s alpha for anxiety were good (alpha = 0.828; alpha = 0.808; alpha = 0.868) and for depression in this study were good to excellent (alpha = 0.872; alpha = 0.869; alpha = 0.920) for Boston, Toronto, and Montréal respectively.

#### Multidimensional Scale of Perceived Social Support

The MSPSS [32] is a twelve-item self-report measure of social support inclusive of three subscales (family, friends, and significant others). Two items from each of the two subscales (family and friends) were used in the current study. Respondents rate their agreement to four statements on a seven-point Likert scale, ranging from 1 (strongly disagree) to 7 (strongly agree). A sample item is, “my family really tries to help me.” A mean score for the four items was calculated with higher scores indicating more social support from family/friends. The MSPSS has demonstrated reliability and validity [10].

### Procedures

#### Boston and Toronto

All procedures were performed in accordance with the 1964 Helsinki declaration and its later amendments or comparable ethical standards; the Institutional Review Board of Boston Children’s Hospital and the REB of Carlton University approved the SYLS. In SYLS, informed consent was obtained by Somali study staff and quantitative interviews were administered verbally in English by non-Somali research staff. Participants were paid $60 as a thank you for their time.

#### Montréal

The study protocol and procedures were approved by the Ethics Committee of the Centre Integré Universitaire de Santé et de Services Sociaux du Centre-Ouest-de-l’Ile-de-Montréal (CIUSSS-CODIM). In addition, the research ethics board of each college gave approval prior to data collection. Researchers uploaded the questionnaire on an intranet portal used by colleges to communicate with students and remained online for a month. Participants completed the survey in either French or English, depending upon their preference. The project was described as a research study on adaptation to the current social context in the province of Québec (Canada). Students were informed that their involvement was voluntary and that their responses would be confidential. Students consented to be part of the study on the first page of the survey. Participants were able to discontinue the survey at any time. Contact information of research team and ethics board members were made available to answer any questions or concerns regarding the study.

### Data Analyses

First, descriptive analyses were conducted. Table 1 provides demographic information and means of variables of interest of study participants by location.

**TABLE 1:** Demographic information of study participants by location.

Characteristics	Total (N = 791)	Montréal (n = 593)	Toronto (n = 95)	Boston (n = 103)
	N (%) or mean ± SD (Range)^a^	N (%) or mean ± SD (Range)^a^	N (%) or mean ± SD (Range)^a^	N (%) or mean ± SD (Range)^a^
Gender				
Male	297 (37.6)	181 (30.6)	60 (63.2)	56 (54.4)
Female	492 (62.4)	410 (69.4)	35 (36.8)	47 (45.6)
Age				
18	12 (1.5)	0 (0.0)	0 (0.0)	10 (9.7)
19–21	503 (63.6)	399 (67.6)	2 (2.1)	40 (38.8)
22–24	157 (19.8)	105 (17.8)	64 (67.4)	27 (26.2)
25–27	74 (9.4)	53 (9.0)	25 (26.3)	17 (16.5)
28–30	39 (4.9)	33 (5.6)	4 (4.2)	6 (5.8)
31+	3 (0.4)	0 (0.0)	0 (0.0)	3 (2.9)
Immigration/generation status				
Non-immigrant	360 (46.1)	360 (60.7)	0 (0.0)	0 (0.0)
1st or 2nd generation	421 (53.9)	223 (37.6)	95 (100)	103 (100)
Region of the world participants (or their parent) were born				
Canada/North America	387 (48.9)	387 (65.3)	0 (0.0)	0 (0.0)
North africa/Middle east	69 (8.7)	69 (11.6)	0 (0.0)	0 (0.0)
Sub-saharan africa	217 (27.4)	19 (3.2)	95 (100)	103 (100)
South America	27 (3.4)	27 (4.6)	0 (0.0)	0 (0.0)
Caribbean	39 (4.9)	39 (6.6)	0 (0.0)	0 (0.0)
Europe	52 (6.6)	52 (8.8)	0 (0.0)	0 (0.0)
HSCL: Anxiety	1.48 ± 0.51 (1–4)	1.58 ± 0.55 (1–4)	1.31 ± 0.38 (1–2.70)	1.24 ± 0.34 (1–2.80)
HSCL: Depression	1.56 ± 0.59 (1–4)	1.74 ± 0.64 (1–4)	1.38 ± 0.41 (1–3.38)	1.29 ± 0.36 (1–2.71)
MSPSS: Social support	5.51 ± 1.29 (1–7)	5.28 ± 1.33 (1.25–7)	6.10 ± 0.95 (2.25–7)	5.86 ± 1.18 (1–7)
EDD: Discrimination	0.45 ± 0.35 (0–1)	0.37 ± 0.34 (0–1)	0.72 ± 0.27 (0–1)	0.60 ± 0.29 (0–1)
RIS: Radical intentions	2.76 ± 1.63 (1–7)	2.60 ± 1.51 (1–7)	3.23 ± 1.90 (1–7)	2.94 ± 1.71 (1–6.40)

Note. EDD, everyday discrimination scale; HSCL, hopkins symptoms checklist; MSPSS, multidimensional scale of perceived family and friend social support; RIS, radicalism intention scale. Data from Wave 2 of the Somali Youth Longitudinal Study (Boston, USA and Toronto, Canada; 2014–2015) and from a multi-site college study (Quebec, Canada; 2016–2017).

^a^ Column displays frequencies and percentages for categorical variables and mean/SDs and range for continuous variables.

#### Multiple Regression Analysis

Data were analyzed by means of a multiple predictors linear regression analysis model using maximum likelihood estimation and the engagement of robust standard errors due to accounting for not meeting normality assumptions. Missing data ranged between 10.7 and 29.8%. Listwise deletion was used to engage only full cases. To ensure that listwise deletion did not result in biased point and variance estimates, means/SDs were estimated using full data and the sample involving full cases only. Results indicated minuscule differences in the estimates of the two datasets: Anxiety (M_full_ = 1.4773, M_Listwise_ = 1.4753), Depression (M_full_ = 1.5951, M_Listwise_ = 1.5883), Radicalism (M_full_ = 2.7608, M_Listwise_ = 2.7778), social support (M_full_ = 5.5086, M_Listwise_ = 5.5896), and discrimination (M_full_ = 0.4510, M_Listwise_ = 0.4645). Consequently, it was concluded that listwise deletion did not result in distorted point estimates. Model fit is not evaluated using omnibus criteria as it is a saturated model; instead, each predictor is evaluated for significance using partial regression coefficients, accounting for the presence of all other predictors in the model. The level of significance was set to 5% for a two-tailed test, in light of the power estimation shown below. Further between-group comparisons were made by use of the Wald test, through specifying equivalence constraints across partial regression coefficients in two groups at a time. All analyses were conducted using Mplus 8.5.

#### Power Analysis

Power for a linear regression model was estimated using six independent variables for the prediction of radicalism. Using a medium effect size of a multiple correlation equal to 0.15, power levels equal to 80% and a two-tailed test using a nominal alpha level of 5%, a sample size of 97 full cases would achieve power levels equal to 80% [33]. We further explored power by estimating the required sample size to estimate as significant standardized slopes equal to 0.3, representing medium-level effects [33]. Using a Monte Carlo simulation positing standardized slopes equal to 0.30 with sample sizes of *n* = 94 (representing the smallest group) and 1,000 replicated samples, results indicated that power levels of the 0.3 slope coefficients were equal to 81.3% with mean coverage levels equal to 94.8% (in the 1,000 replicated samples). Consequently, the present study had ample levels of power for evaluating the predictive ability of these linear predictors.

## Results

Table 2 presents intercorrelations between measured variables across locations. Notable correlations were a strong positive relationship between anxiety and depression across locations (ranging between r = 0.710 and r = 0.797), the positive correlation between discrimination and anxiety/depression across locations (ranging between r = 0.235 and r = 0.351), and a negative relationship between age and radicalism consistently across areas (ranging between −0.162 and −0.294). The remaining relationships varied across locations; for example, a negative relationship between social support and discrimination was observed for Montréal and Toronto (ranging between r = −0.178 and r = −0.187) but was non-significant in the Boston area. Similarly, social support related negatively with anxiety and depression in Montréal and Toronto (ranging between r = −0.182 and r = −0.381), but not Boston (where only depression was significant).

**TABLE 2:** Intercorrelations between measured variables by location.

Variables	Sex	Age	Anxiety	Depression	Social Support	Discrimination	Radicalism
**Montréal**							
Sex	1						
Age	0.051	1					
Anxiety	0.162**	0.033	1				
Depression	0.178**	0.001	.710**	1			
Social Support	−0.079	0.001	−.182**	−.327**	1		
Discrimination	0.031	0.043	.263**	.344**	−0.178**	1	
Radicalism	−0.246**	−0.162**	−0.008	0.078	0.01	0.002	1
**Toronto**							
Sex	1						
Age	−0.016	1					
Anxiety	0.331**	−0.219**	1				
Depression	0.342**	−0.178	0.797**	1			
Social Support	−0.079	−0.029	−0.363**	−0.381**	1		
Discrimination	−0.072	−0.095	0.266**	0.235*	−0.187	1	
Radicalism	0.103	−0.208*	0.154	0.137	−0.282*	0.212*	1
**Boston**							
Sex	1						
Age	−0.115	1					
Anxiety	0.016	−0.073	1				
Depression	0.036	0.099	0.774**	1			
Social Support	−0.019	−0.257**	−0.091	−0.360**	1		
Discrimination	−0.178	−0.03	0.258**	0.351**	−0.123	1	
Radicalism	−0.163	−0.294**	0.147	0.065	0.187	0.087	1

Note. *p < 0.05; **p < 0.01. Data from Wave 2 of the Somali Youth Longitudinal Study (Boston, USA and Toronto, Canada; 2014–2015) and from a multi-site college study (Quebec, Canada; 2016–2017).

### Prediction of Violent Radicalization Using Radical Intention Scores From Personal Characteristics

**FIGURE 1:**
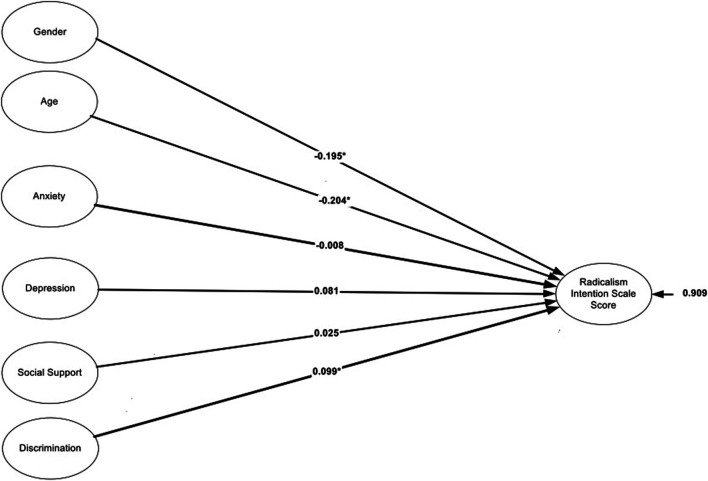
Prediction of radicalism intention scale score from demographics and psychological measures for the full data. Data from Wave 2 of the Somali Youth Longitudinal Study (Boston, USA and Toronto, Canada; 2014–2015) and from a multi-site college study (Quebec, Canada; 2016–2017).

Figure 1 displays the findings from using the full sample (i.e., aggregating data across all locations). As shown in the figure, among predictors of radical intention scores (RIS), those that exceeded conventional levels of significance were gender, age, and level of discrimination. Concerning gender, being a female was associated with significantly lower scores on the RIS compared to being a male (*b* = −0.195, *p* < 0.05) and older individuals had lower RIS scores (*b* = −0.204, *p* < 0.05). With regard to discrimination, the higher the levels of discrimination the higher the scores on the RIS (*b* = 0.099, *p* < 0.05). The total amount of variance of radicalism predicted by the linear combination of the independent variables was 6%, significantly different from zero. This effect represents a medium effect size based on [33] suggestions of medium level predictions using r-square related indices (i.e., small = 0.01, medium = 0.06, large = 0.14).

### Moderated Regression Predicting Violent Radicalization From Personal Characteristics by Area

**FIGURE 2:**
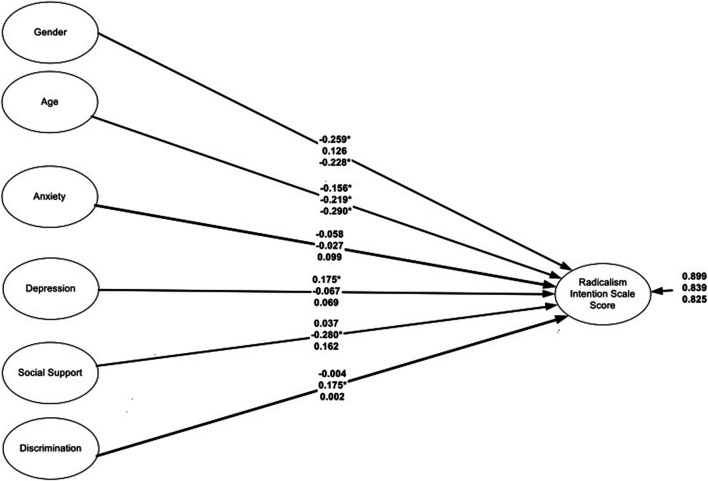
Prediction of radicalism intention scale score from linear predictors by location. Coefficients are standardized. The order of the coefficients is as follows first = Montréal, middle = Toronto, last = Boston. Data from Wave 2 of the Somali Youth Longitudinal Study (Boston, USA and Toronto, Canada; 2014–2015) and from a multi-site college study (Quebec, Canada; 2016–2017).

Table 3 displays the findings from the moderated regression analysis using location as the grouping variable (see also Figure 2). Being a female was associated with lower radicalism scores in Montréal compared to Toronto (Diff_Slope_ = −0.39, *p* < 0.05) and in Boston in relation to Toronto (Diff_Slope_ = −0.35, *p* < 0.05). Age was not a significant moderator as its negative propensities in predicting radicalism were consistent across locations. Similarly, there were no moderated effects due to anxiety, which was consistently not predictive of levels of radicalism across all locations. The moderated effects of depression were evident as it exerted positive effects on radicalism using the Montréal only sample; the effects of depression in Toronto and Boston were null. Social support exerted negative effects on radicalism in Toronto only (b = −0.28) with the respective slopes in Montréal and Boston being non-significant. Last, discrimination was a significant moderator as its effects were significantly more pronounced in Toronto compared to Montréal and Boston. Specifically, the higher the levels of discrimination the higher the levels of radicalism in the Toronto area. All other effects were null.

**TABLE 3:** Comparison between regression coefficients across locations using the wald test.

	Standardized coefficient			Slope coefficient	Wald test	*p*-value
Predictors	Montréal	Toronto	Boston	Comparison		
Gender	−0.259*	0.126	−0.228*	Montréal vs. Toronto	9.559	0.002*
				Montréal vs. Boston	0.081	0.776
				Toronto vs. Boston	6.347	0.012*
Age	−0.156*	−0.219*	−0.290*	Montréal vs. Toronto	1.773	0.183
				Montréal vs. Boston	1.052	0.305
				Toronto vs. Boston	0.803	0.370
Anxiety	−0.058	−0.027	0.099	Montréal vs. Toronto	0.002	0.963
				Montréal vs. Boston	0.609	0.435
				Toronto vs. Boston	0.332	0.564
Depression	0.175*	−0.067	0.069	Montréal vs. Toronto	0.822	0.365
				Montréal vs. Boston	0.001	0.987
				Toronto vs. Boston	0.384	0.535
Social support	0.037	−0.280*	0.162	Montréal vs. Toronto	10.280	0.001*
				Montréal vs. Boston	2.634	0.105
				Toronto vs. Boston	11.863	0.001*
Discrimination	−0.004	0.175*	0.002	Montréal vs. Toronto	3.521	0.061
				Montréal vs. Boston	0.014	0.906
				Toronto vs. Boston	2.068	0.150

Note. Wald tests contrast regression coefficients between locations. For example, the effects of gender were significantly different between Montréal and Toronto (*b*_Montréal_ = -0.259, *p* < 0.05; *b*_Toronto_ = 0.126, *p* = n.s.) as pointed out by the significant Wald statistics (Wald = 9.559, *p* < 0.05). Data from Wave 2 of the Somali Youth Longitudinal Study (Boston, USA and Toronto, Canada; 2014–2015) and from a multi-site college study (Quebec, Canada; 2016–2017).

## Discussion

Overall, our findings highlight the importance of considering contextual differences in risk and protective factors associated with support of VR. When data from the three cities were pooled, discrimination, age, and gender were associated with support of VR. However, each of the three cities provided different pictures of the relative importance of various risk and protective factors in relation to support of RV.

In Montréal the predominance of depression as a risk factor for a mixed majority-minority sample may reflect the growing feelings of helplessness and insecurity of Montréal youth, which have been documented repeatedly [34], and its association with a negative view of the future [35]. For this generation, the adoption of a dystopian view of life seems to be a strategy to confront the uncertain future and represent the existential doom associated with it (Venkatesh et al, 2019). Thus the endorsement of attitudes legitimizing violence by depressed youth can be seen as a cultural shift in idioms of distress associated with pain and despair, just as, in previous generations, self-mutilation has become a challenge to security oriented societies [36]. The relatively smaller portion of minorities in this sample may also lessen the importance of discrimination as a risk factor. Furthermore, college students may differ from the young adults included in the Boston and Toronto samples (which included both students and non-students) in other ways, such as a sense of opportunity, which may also have contributed to the diminished role of discrimination.

In Toronto discrimination and social support both significantly predicted support of VR. The potency of these particular variables in our Toronto sample, which consists of ethnic Somalis, may highlight the way in which belonging to an ethnic minority group in a multicultural society can offer both risk and protection. A greater openness to the use of violence among those who experienced high levels of discrimination may be a more externalized response to suffering, and one that on some level reflects anger and a lack of complacency in accepting marginalization as the status quo. Connection to community, in contrast, may serve to buffer a sense of marginalization and is associated with reduced support for attitudes that legitimize violence. Notably, gender did not predict support of VR, a finding that may be explained by the salience of grievances resulting from a sense of injustices perpetuated against a vulnerable community by powerful institutions such as law enforcement and the media. Such powerful grievances may be common across males and females, and supercede potential gender differences.

There are a number of reasons, both methodological and theoretical, that may explain variation in findings based on location. First, the differences are likely a reflection of the populations drawn on for this pooled dataset. While differences in method and population cannot be ignored, if differences between models were due solely to this than findings from Toronto and Boston should have been similar. The fact that the Toronto model demonstrated significant associations between support for VR and both discrimination and low social belonging, while neither of these factors was significant in the Boston sample, suggests that regional differences remain even when study methods and ethnic composition of samples is the same.

Second, findings may be a reflection of regional sociopolitics or cultural discourse related to issues such as discrimination or mental health, and cultural idioms of distress. Sociopolitical or cultural differences within the various regions may contribute to varying levels of comfort in reporting different variables. Furthermore, sociopolitical differences between different regions may lead to fundamentally distinct experiences which, in turn, leads to different forces shaping support of VR. Within Toronto, discrimination was not only highly predictive of support of VR, but was also more prevalent compared to levels experienced by the same ethnic community in Boston. An important question to further explore is whether discrimination is such a potent risk factor in Toronto in part due to the more ubiquitous nature of such experiences.

Another possibility is that the discourse within Toronto and Boston on Somalis and radicalism differs as a result of recent sociopolitical events, and this in turn shapes the risk factors for radicalism. The focus on the Somali community in Toronto related to the “Toronto 18,” vs the “Boston Strong” discourse that followed the Boston marathon bombings may have led to different contexts of perceived safety among Somalis when talking about radicalism. In a context of perceived safety, hypothesized under this framing to be more salient in Boston, endorsement of support for VR on a questionnaire may reflect an underlying comfort and stability, rather than grievance. In this case, variables reflecting distress or adversity (e.g. discrimination or depression) may be less expected to relate to higher levels of support of radicalism. In contrast, in a setting where mention of radicalism carries with it overtones of threat and stigma, support of radicalism may be a reflection of high distress as opposed to comfort and stability. Further work understanding local strains and distress, and how local media or events may create conditions of perceived safety or threat, may help to elucidate additional variables that should be included to capture regional experiences that may relate to support of VR.

### Limitations

There are several limitations to this study. First, data is cross-sectional and we cannot make causal inferences about the relationship between study variables. Cross-site comparisons are also limited given differences in study populations and size, particularly when contrasting Montréal, comprised of a racially and ethnically diverse sample of college youth, and Toronto/Boston, comprised of Somali immigrant young adults. Data collection procedures also differed significantly between studies; in particular, the use of an on-line survey in Montréal resulted in low response rate and a study sample that may not be representative of the larger city population. A further methodological difference is that the SYLS used a modified version of the RIS; although this version demonstrated comparable psychometrics, it is likely that the two versions led to different rates of endorsement. An additional limitation is that the variable assessing discrimination did not include structural or systemic racism.

## Public Health Implications and Conclusions

This study raises questions about the generalizability of findings related to risk and protective factors for support of VR from one geographical setting and/or population to others. In the broader field of violence prevention, it is well established that local contexts influence patterns of interpersonal violence [37]. Integrating meso and macro-level predictors of support of VR into public health practice entails developing and/or adapting existing primary prevention initiatives and policies to acknowledge and address broader contextual issues that influence support for VR. Although the need for primary prevention initiatives based on a resilience-oriented socio-ecological framework is increasingly emphasized by scholars [15], examples and empirical evaluations of such primary prevention programs are scarce and highlight that they lead to negative outcomes when they targeted specific ethnic, racial or religious groups [38, 39]. At present, most policies and interventions focus on building resilience against the influence of extremist propaganda and narratives in young and vulnerable individuals, failing to address systemic injustices, discrimination, polarized political discourses and violence in our societies [39]. Our findings suggest that primary prevention initiatives should consider that the source of adversity and violence can reside within the social order of a specific local context and thus necessitates adaptation across contexts. Bridging VR prevention policies and social policies aimed at promoting inclusion and social justice, empowering individuals and communities at a local level in a bottom-up approach is a promising way to move forward.

## Data Availability

The datasets presented in this article are not readily available because data analyzed in this study are pooled from multiple datasets. Requests to access the datasets should be directed to the Trauma and Community Resilience Center at Boston Children's Hospital, tcrc@childrens.harvard.edu.
